# Frontal Theta Oscillation as a Mechanism for Implicit Gender Stereotype Control: Electrophysiological Evidence From an Extrinsic Affective Simon Task

**DOI:** 10.3389/fnhum.2020.573187

**Published:** 2020-12-17

**Authors:** Lei Jia, Mengru Cheng, Billy Sung, Cheng Wang, Jun Wang, Feiming Li

**Affiliations:** ^1^Department of Psychology, Zhejiang Normal University, Jinhua, China; ^2^School of Marketing, Curtin University, Perth, WA, Australia

**Keywords:** stereotype control, neural oscillation, frontal midline theta (FMθ), time-frequency analysis, extrinsic affective Simon task (EAST), event-related potential (ERP)

## Abstract

Previous research has indicated that frontal midline theta (FMθ) reflects a domain-general cognitive control mechanism of the prefrontal cortex. Brain imaging studies have shown that the inhibition of implicit stereotypes was dependent on this domain-general cognitive control mechanism. Based on this knowledge, the present study investigated the neural oscillatory correlates of implicit gender stereotype control in an extrinsic affective Simon task (EAST) using electrophysiological methods. Participants in this task conducted verification to white gender names and colored gender traits, and their behavioral response and electroencephalogram (EEG) were recorded during their performances. As predicted, stereotype-inconsistent trials resulted in reduced response accuracies and slower response times than stereotype-consistent trials. For the event-related potential (ERP) results, the enhanced performance of stereotype-inconsistent trials was accompanied by an enhanced N400 amplitude but an attenuated late positive potential amplitude. In contrast, early attentional components such as P2 and N2 as well as their amplitudes were impacted by the experimental manipulations and individual differences in gender factors. In addition, based on time–frequency (TF) analysis, we found that the enhanced performance of stereotype-inconsistent trials was also accompanied by an event-related synchronization on the frontal theta oscillation. This frontal theta appeared at a late processing stage and persisted across a time window from N400 to late positive potential. Additionally, this enhanced frontal theta effect was not modulated by the experimental manipulations and individual differences in gender factors. Based on these findings, the feature of frontal theta oscillation in the implicit gender stereotype control process was discussed.

## Introduction

In social perceptions, activation of group categories usually leads to heightened accessibility of stereotype (Macrae and Bodenhausen, 2000). Cognitive control is essential to avoid the unwanted influence of stereotype activation and to overcome conflicts caused by stereotype violation (Devine, 1989; Amodio et al., 2008). Cognitive control is a complex but crucial capacity that consists of different components, among which conflict monitoring and response inhibition are two important examples (MacDonald et al., 2000; Botvinick et al., 2001). In the past few decades, a number of reports have conceptualized performance in stereotyping tasks in terms of conflict and control processes. Control of stereotypes has also been considered as a part of a more general skillset associated with effective self-regulation, requiring implementation of top-down control over well-learned, automatically activated stereotypical belief and situational-inappropriate responses (Bartholow and Dickter, 2008).

Compared to the general conflict controls, stereotype control is also largely seen as rooted in dual-process models (i.e., the automatic and control processes; Devine, 1989; Blair and Banaji, 1996; Moskowitz and Li, 2011), particularly on the importance of conflict monitoring and response inhibition in social judgments and social behaviors. These dual-process models are usually utilized to describe the processes of conflict control, in which a first set of processes inexorably gives rise to specific outputs (e.g., implicit/automatic stereotype activation). However, a subsequent or second set of processes may be incompatible with those outputs and draw on conscious control to inhibit the first outputs from influencing the ultimate response of the individual (e.g., stereotype control, which prevents the activated stereotype). Based on this theoretical frame, stereotype control is usually regarded as a cognitive control component that inhibits the activated stereotypical belief and situational-inappropriate responses at the late temporal processing stage of stereotyping (Devine, 1989; Blair and Banaji, 1996; Moskowitz and Li, 2011).

For example, in stereotype control studies using conflict control paradigms [e.g., implicit association test (IAT), Go/No-Go, and their variations such as weapons/tool and kitchenware/tool identification task], participants usually respond more slowly to stereotype-inconsistent (SI) conditions than stereotype-consistent (SC) or control conditions (Bartholow et al., 2006; Correll et al., 2006; Dickter and Bartholow, 2007; Knutson et al., 2007; Amodio et al., 2008; Ma et al., 2008; White et al., 2009; Cattaneo et al., 2011). In line with this effect, the control of stereotypes in that situation would result into a greater activation of cognitive control regions in prefrontal cortices, particularly in the dorsolateral prefrontal cortex (DLPFC), the anterior cingulate cortex (ACC), and the medial prefrontal cortex (MPFC; Knutson et al., 2007; Cattaneo et al., 2011). Simultaneously, conflict-related brain potentials such as N2 [including correct-response negativity (CRN) and error-related negativity (ERN)], P3, NSW, N400, and late positive potentials (LPPs) were elicited and enhanced at a late temporal processing stage and showed their susceptibility to conflicts caused by stereotype violation Bartholow et al., 2003, 2006; Correll et al., 2006; Dickter and Bartholow, 2007; Amodio et al., 2008; Ma et al., 2008; White et al., 2009; Jia et al., 2012). Taken together, all existing evidence supports the hypothesis that stereotype control has a similar conflict interference mechanism with domain-general cognitive control.

In previous electrophysiological studies on domain-general cognitive control, an important research approach is spectral (i.e., neural oscillation) analysis, which can estimate the amount of neural activity in particular frequency bands. Recently, an important finding from this research approach is that frontal midline theta (FMθ) may serve as a marker for cognitive controls (Cavanagh and Frank, 2014; Brunetti et al., 2019; Nigbur et al., 2011; Liu et al., 2014; Padrão et al., 2015). The theta rhythm of human scalp electroencephalogram (EEG) is commonly defined at 4–8 Hz. It is principally generated in structures of the limbic system such as the hippocampus and the cingulated cortex, but it is also found in the prefrontal cortex (Nigbur et al., 2011). In previous research, theta rhythm has been acknowledged for a wide span of cognitive functions such as focused attention, novelty encoding, working memory loading, semantic priming, and top-down cognitive control (Cavanagh and Frank, 2014). In particular, for its cognitive control role in conflict monitoring and resolution task, time–frequency (TF) analyses have suggested that the FMθ activities recorded from sensors overlying MPFC/ACC could be quantified as N2, ERN/CRN component. Taken together, it may reflect enhanced demands in cognitive control and performance monitoring (Nigbur et al., 2011; Cavanagh and Frank, 2014).

Although a number of previous studies have identified the N2/ERN, P3, NSW, N400, and LPP component as conflict processes for stereotype control (e.g., Bartholow et al., 2006; Dickter and Bartholow, 2007; Amodio et al., 2008; Bartholow and Dickter, 2008; White et al., 2009; Jia et al., 2012), to our knowledge, only a few of them reported the neural oscillatory correlates of stereotype control. Consequently, it remains unknown whether the FMθ that indexed the domain-general cognitive control would appear in stereotype control tasks. Hence, the main intention of the present study was to examine and address this literature gap.

To achieve this, in the current study we employed a specialized conflict control paradigm [i.e., the extrinsic affective Simon task (EAST)], to induce the control processes of the implicit gender stereotype. The EAST can be regarded as a variant of the IAT, which is widely used in implicit social cognition measurement. Similar to the IAT, implicit social cognition in this task is also measured through two categorization tasks (i.e., the target and its label property/valence). But a feature of the EAST is that it mixes the categorization task on label properties (or valence) with a color judgment (usually green or blue colors) on the targets. This feature allows the two association tasks in IAT to be combined as a single EAST task, whereby the beliefs toward the targets could be measured on the basis of the implicit automatic association between targets and their label properties. Therefore, the EAST can optimize and simplify the procedures to measure implicit stereotyping. Additionally, another advantage of the EAST over the IAT is that it is less susceptible to the nonassociative effects of task recoding, and this paradigm could be used to assess the control or suppression processes. Therefore, this paradigm has been frequently used in research on cognitive/affective expression and inhibition processes (De Houwer, 2006; Wentura, 2010).

**Figure 1:**
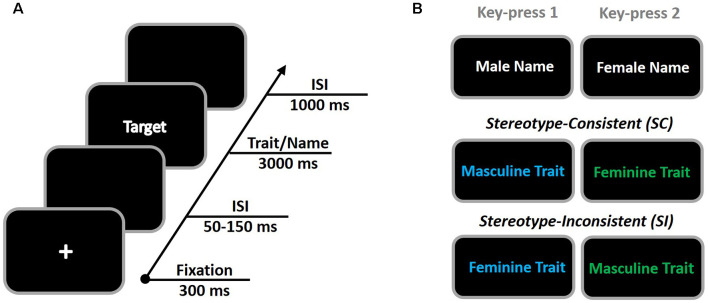
The procedure of the extrinsic affective simon task (EAST; **A**) and its stimuli illustration **(B)**. When a colored trait was presented, participants were instructed to press the key with their right hand on the basis of its color (blue = key 1, green = key 2). Alternatively, when a white name was presented, participant was instructed to press keys with their right hand to indicate the gender of the name (male = key 1, female = key 2). Using the consistent and inconsistent gender “name-trait” association in EAST, stereotype-consistent and stereotype-inconsistent processes were evoked.

In the current EAST task, we utilized gender names and gender traits, respectively, as targets and their label properties (Figure 1). All gender traits were presented in two colors (blue and green for each), whereas the gender names were presented in white text. Correspondingly, we intended to induce the implicit gender stereotyping by means of the automatic semantic associations between gender names and gender traits. This paradigm ensured a current experiment design as 2 (stereotype compatibility: consistent/inconsistent) × 2 (gender preference: masculine/feminine) × 2 (gender of participant: male/female). With EEG measurement, we tested two hypotheses in the present study. First, as the implicit gender stereotypes were manipulated in EAST, we would like to test the temporal processing characters of gender stereotyping using traditional event-related potential (ERP) analysis. Like the general conflict control paradigms (e.g., Stroop task, Go/No-Go), this EAST task could also be divided into two processing stages based on dual-process models: an early automatic semantic stage for gender stereotype activation and, subsequently, a late controlled stage for stereotype control (van Veen and Carter, 2005). Compared to the semantic stage for gender stereotype activation, here we predicted that the stereotype control processes would happen at the late temporal processing stage. Specifically, we expect to see increased activities of late ERP components that are sensitive to the stereotype control process (e.g., N400 or LPP). Second, we would like to test the hypothesis that the FMθ of the top-down domain-general cognitive control would reflect the stereotype control process in the EAST. If so, we would observe an enhanced power of FMθ in the SI condition when compared to SC condition at a late temporal processing stage.

## Materials and Methods

### Subjects

The present research was approved by the research ethics committee of the Zhejiang Normal University. A total of 36 undergraduates (19–24 years, 19 male and 17 female participants) were recruited through advertisements. All participants were right-handed and reported no neurological disorders, significant physical illness, head injury, or alcohol/drug abuse. The participants provided their written informed consent to participate in this study. After the experiment, they were debriefed, thanked, and paid RMB¥40 as a compensation for participating. Two female subjects were excluded in the former data analysis because the reference channel of their EEG recording produced poor quality of data.

### Materials

Experimental materials included typical Chinese gender names and supposedly typical gender traits (all were made by two Chinese characters; see Supplementary Table 1 in Supplementary Materials). Gender names were made up of 12 typical Chinese male names and 12 typical Chinese female names. These gender names were familiar among all participants. Correspondingly, 12 positive masculine traits (e.g., heroic, intrepid, aggressive) and 12 positive feminine traits (e.g., attractive, lovely, elegant) were chosen from a gender role scale. Their gender role scales were validated on an independent sample of 37 volunteers to ensure that they were consistent with the general male and female stereotypes in Chinese culture. On a nine-point scale (1 = very masculine, 5 = gender neutral, 9 = very feminine), the masculine traits had an average value of 2.03 (SD = 0.37) and feminine trait had an average value of 7.96 (SD = 0.38), which were significantly different from one another, *F*_(1,23)_ = 1,458.05, *p* < 0.001, $\eta_{\text{p}}^{2}$ = 0.89. The same traits were rated on a five-point scale (gender neutral to gender specific: 0 = nonsexual, 4 = very sexual), and no significant difference emerged between the masculine (mean = 2.98, SD = 0.37) and feminine traits (mean = 2.86, SD = 0.38), *F*_(1,23)_ = 0.57, *p* > 0.05. As targets in the EAST, those masculine and feminine traits and names were randomly presented in the experiment.

### Procedures

Referring to Figure 1, the procedure of the EAST in the present study was adopted from prior research (i.e., De Houwer, 2006). All masculine and feminine traits were presented in two colors (blue and green for each), whereas the names were presented in white text. All stimuli were presented randomly in the center of a black screen with 36-point Chinese Song character font. Participants were told to classify target words by pressing different keys depending on their meanings or colors. When a colored trait was presented, participants were to press the key with their right hand on the basis of its color (blue = key 1, green = key 2). Alternatively, when a white name was presented, participants were to press keys with their right hand to indicate the gender of the name (male = key 1, female = key 2).

The whole task involved 288 trials, including 144 name trials and 144 trait trials. All trials were randomly presented in three blocks. Half of the 144 trait judgments represented the consistent trials, whereas the other half were inconsistent trials. For the consistent trials (i.e., blue-colored masculine traits and green colored feminine traits), participants’ key-pressing is consistent between the color and gender trait. Thus, these trials served as the SC condition and indexed the stereotype expression process. For the inconsistent trials (i.e., green colored masculine traits and blue-colored feminine traits), participants’ key-pressing is inconsistent between color and gender trait. Thus, these trials served as the SI condition and indexed the stereotype inhibition processing.

### EEG Recording and Preprocessing

EEG data were recorded at 64 scalp sites using tin electrodes mounted on an elastic cap (Brain Product GmbH, Munich, Germany), with the reference electrode on the right mastoid and the ground electrode on the FCz. The horizontal electro-oculogram was recorded with electrodes placed outside the eyes, and the vertical electro-oculogram was recorded with electrodes placed above and below the left eye. All interelectrode impedances were maintained below 5 μV. The EEGs and electrooculograms (EOGs) were amplified using a 0.01- to 100-Hz bandpass filter and continuously sampled at 500 Hz.

After recording, EEG data were preprocessed using the EEGLAB 13.7 (Swartz Center for Computational Neuroscience; Delorme and Makeig, 2004) on MATLAB software. EEG data were 0.5- to 30-Hz filtered and then segmented from −1,000 to 2,000 ms around trait onset, with the average of the left and right mastoids as the off-line reference. Next, eye movement artifacts (e.g., eye blinks, muscle artifacts) were removed by using independent component analysis. Finally, trials with error responses and those contaminated with artifacts due to amplifier clipping, bursts of electromyographic activity, or peak-to-peak deflection exceeding ±80 μV were excluded. That reserved a total of 90% trials and more than 20 trials at each condition for each participant.

### ERP Analysis

**Figure 2:**
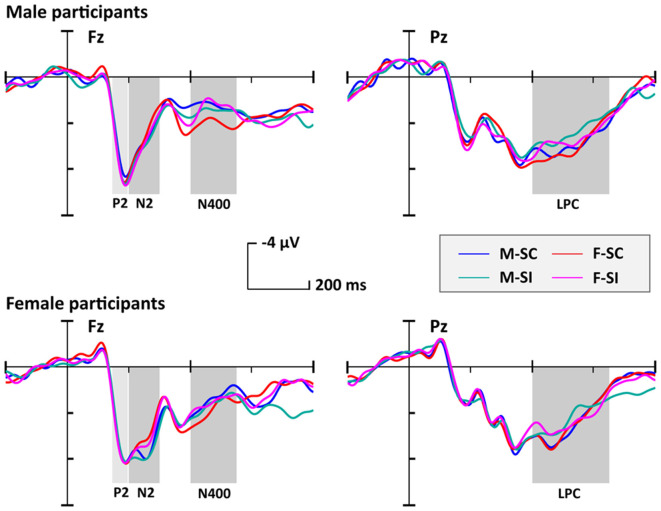
The grand-averaged event-related potentials (ERPs) for each condition. Each male or female participant needed to judge four trait conditions, respectively, as (1) masculine-stereotype-consistent (M-SC), (2) feminine-stereotype-consistent (F-SC), (3) masculine-stereotype-inconsistent (M-SI), and (4) feminine-stereotype-inconsistent (F-SI).

**Figure 3:**
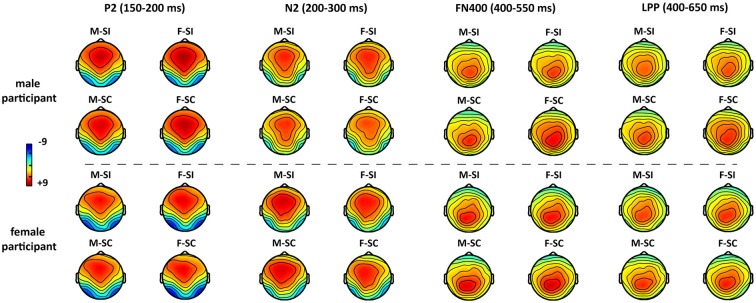
The topographical distribution of P2, N2, N400, and LPP for each condition. Abbreviations: masculine-stereotype-consistent (M-SC), feminine-stereotype-consistent (F-SC); masculine-stereotype-inconsistent (M-SI), and feminine-stereotype-inconsistent (F-SI).

The averaged epochs for ERP measurement were based on the participants’ mean response times (RTs). Consequently, a 1,000-ms time window including 200 ms per target (baseline) and 800 ms posttarget was used in the EEG overlay and ERP analysis. The grand-averaged ERPs (Figure 2) showed that the processing of gender traits mainly elicited early perception components such as N1 and P2, as well as conflict-control–related components such as N2, N400, and LPP. In particular, the P2, N2, and N400 components had topographical distributions at anterior/frontal scalp, whereas the LPP had a posterior topographical distribution (Figure 3).

To test our hypotheses, we focused on the P2, N2, N400, and LPP components, which are associated with gender categorization or stereotype control in previous studies (Bartholow et al., 2006; Dickter and Bartholow, 2007; Amodio et al., 2008; Bartholow and Dickter, 2008; White et al., 2009). Based on their grand-averaged waves and topographical distributions, we selected a set of 10 anterior–center electrodes (i.e., Fz/F1/F2/F3/F4/FC1/FC2/Cz/C1/C2) as regions of interest (ROIs) for the P2, N2, and N400 average amplitude analysis. In addition, we also selected a set of 11 posterior–center electrodes (i.e., Cz/C1/C2/CPz/CP1/CP2/Pz/P1/P2/P3/P4) for the LPP average amplitude analysis. A mixed-model analysis of variance (ANOVA) with two within-subject factors [i.e., stereotype compatibility (SC vs. SI) and gender preference of traits (masculine vs. feminine)] and one between-subject factor [participant’s gender (male vs. female)] was conducted on the average amplitudes of P2, N2, N400, and LPP components. *p*-values were corrected by Greenhouse–Geisser correction.

### TF Analysis

To measure the event-related synchronization or desynchronization (ERS and ERD) of neural oscillations associated to the stereotype processes, trial-by-trial event-related spectral power modulations of the EEG rhythms were computed using the “*newtimef*” function of the EEGLAB. A version of Hanning-windowed sinusoidal wavelets of three cycles at 3 Hz, rising linearly to about 15 cycles at 30 Hz, was used in the TF decomposition. ERD and ERS power changes (in dB) were indexed by the event-related spectral perturbation (ERSP; Delorme and Makeig, 2004). After that, ERSP results were normalized with a pre-stimulus baseline from −200 ms to 0 before target onset.

The permutation test in ANOVA model was implemented in three steps based on previous research (i.e., Hamm et al., 2011, 2012). Specifically, each TF point was first compared with a mixed-model ANOVA with two within-subject factors [i.e., stereotype compatibility (SC vs. SI) and gender preference of traits (masculine vs. feminine)] and one between-subject factor [participant’s gender (male vs. female)]. These yielded distributions of *F*-values for each TF point of interest and for each effect (i.e., the main effects and interactions). To control for the possibility that large effects were the result of a small number of deviant values, this same set of ANOVA was then run 1,000 times (bootstrap procedure), with each factor randomly shuffled at each step (sampling with replacement). After that, probability estimates of the actual *F*-values were then calculated as the proportion of randomly generated *F*-values greater than the actual estimate. Finally, to control for increased family-wise error rate due to multiple comparisons, a cluster-wise statistical method was used to account for the nonindependence of data from adjacent TF points. As suggested by previous research using Monte Carlo simulations, clusters of TF effect were considered significant at family-wise alpha at *p* < 0.01 if at least eight adjacent TF points were significant at *p* < 0.025 (Hamm et al., 2011, 2012).

## Results

### Behavioral Results

**Figure 4:**
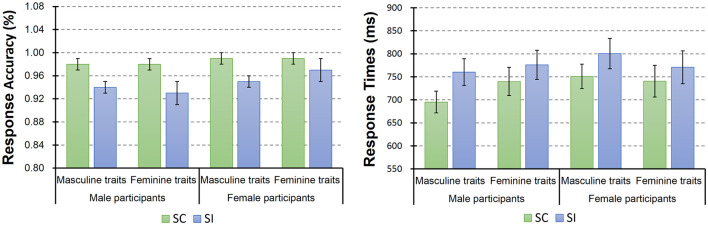
Descriptive statistic results of participants’ task accuracy and response times. The column and bar in this figure denoted the mean and standard error (i.e., mean ± SE), respectively. The statistical analysis on participants’ response accuracy showed a significant main effect of the stereotype compatibility, where SC trials gained higher accuracy than SI trials. In addition, the statistical analysis on response times revealed a significant main effect of the stereotype compatibility and also a significant participant’s gender × trait’s gender preference interaction. *Post hoc* tests showed that participants on average responded significantly faster to the SC trials than the SI trials. Additionally, male participants showed a trend that they responded faster to the masculine traits than feminine traits (see right figure).

Descriptive statistic results of behavioral data are shown in Figure 4. Here we conducted a mixed-model ANOVA with two within-subject factors [i.e., stereotype compatibility (SC vs. SI) and gender preference of trait (masculine vs. feminine)] and one between-subject factor [participant’s gender (male vs. female)]. This ANOVA on response accuracy only revealed a significant main effect of the stereotype compatibility (*F*_(1,32)_ = 30.76, *p* < 0.001, $\eta_{\text{p}}^{2}$ = 0.49), as well as a trend of the participants’ gender main effect (*F*_(1,32)_ = 3.12, *p* = 0.087, $\eta_{\text{p}}^{2}$ = 0.09). However, the main effect of gender preference of trait was not significant (*F*_(1,32)_ = 0.13, *p* = 0.73). *Post hoc* tests showed that SC trials (mean = 98.57%, SE = 0.28%) yielded significantly higher mean accuracies than the SI trials (mean = 94.72%, SE = 0.79%). Besides that, no significant interaction was found.

The ANOVA on RTs also revealed a significant main effect of the stereotype compatibility (*F*_(1,32)_ = 29.49, *p* < 0.001, $\eta_{\text{p}}^{2}$ = 0.48), but the main effects of trait’s gender preference (*F*_(1,32)_ = 0.17, *p* = 0.68) and participant’s gender (*F*_(1,32)_ = 0.32, *p* = 0.57) were not significant. *Post hoc* tests showed that participants on average responded significantly faster to the SC trials (mean = 731.72 ms, SE = 18.93 ms) than the SI trials (mean = 776.81 ms, SE = 22.35 ms). In addition, here we also found a significant participant’s gender × trait’s gender preference interaction (*F*_(1,32)_ = 4.46, *p* = 0.043, $\eta_{\text{p}}^{2}$ = 0.12). For the simple effect of this interaction, male participants showed a trend that they responded faster to the masculine traits than feminine traits, *F*_(1,32)_ = 3.62, *p* = 0.066, $\eta_{\text{p}}^{2}$ = 0.10. Besides that, no significant effect was found.

### ERP Results

#### P2 (150–200 ms)

The ANOVA on P2 amplitude only revealed a significant main effect of the participant’s gender (*F*_(1,32)_ = 4.51, *p* = 0.041, $\eta_{\text{p}}^{2}$ = 0.12), but the main effect of the stereotype compatibility (*F*_(1,32)_ = 0.21, *p* = 0.65) and the main effects of trait’s gender preference (*F*_(1,32)_ = 1.39, *p* = 0.25) were not significant. *Post hoc* tests showed that male participants evoked a larger P2 amplitude (mean = 7.09 μV, SE = 0.76 μV) than female participants (mean = 4.65 μV, SE = 0.86 μV). Besides that, no significant interaction was found (*F*’s < 0.24, *p*’s > 0.63).

#### N2 (200–300 ms)

Analyses on N2 amplitude revealed only a marginally significant main effect of the trait’s gender preference, (*F*_(1,32)_ = 3.33, *p* = 0.077, $\eta_{\text{p}}^{2}$ = 0.09), but the main effect of the stereotype compatibility (*F*_(1,32)_ = 1.01, *p* = 0.32) and the main effect of participant’s gender (*F*_(1,32)_ = 0.04, *p* = 0.85) were not significant. *Post hoc* tests showed that participants evoked a larger N2 amplitude to the masculine traits (mean = 5.48 μV, SE = 0.82 μV) than that to the feminine traits (mean = 4.99 μV, SE = 0.80 μV). Besides that, no significant interaction was found (*F*’s < 1.39, *p*’s > 0.25).

#### N400 (400–500 ms)

Analyses on N400 amplitude only revealed a significant main effect of the trait’s stereotype compatibility (*F*_(1,32)_ = 4.81, *p* = 0.036, $\eta_{\text{p}}^{2}$ = 0.13), but the main effect of the trait’s gender preference (*F*_(1,32)_ = 0.68, *p* = 0.42) and the main effect of participant’s gender (*F*_(1,32)_ = 2.20, *p* = 0.15) were not significant. *Post hoc* tests showed that participants evoked a larger N400 amplitude (mean = 2.96 μV, SE = 0.70 μV) to the SI traits than that to the SC traits (mean = 3.48 μV, SE = 0.66 μV). Besides that, no significant interaction was found (*F*’s < 2.72, *p*’s > 0.11).

#### LPP (400–650 ms)

Similar to the results on N400 amplitude, the ANOVA on LPP amplitude only revealed a significant main effect of the trait’s stereotype compatibility (*F*_(1,32)_ = 5.87, *p* = 0.021, $\eta_{\text{p}}^{2}$ = 0.16), but the main effect of the trait’s gender preference (*F*_(1,32)_ = 0.66, *p* = 0.42) and the main effect of participant’s gender (*F*_(1,32)_ = 1.76, *p* = 0.19) were not significant. *Post hoc* tests showed that participants evoked a larger LPP amplitude (mean = 4.41 μV, SE = 0.54 μV) to the SC traits than that to the SI traits (mean = 3.91 μV, SE = 0.59 μV). Besides that, no significant interaction was found (*F*’s < 0.44, *p*’s > 0.51).

### TF Results

The grounded averages of TF decomposition for each condition are shown in Supplementary Figure 1 in the Supplementary Materials. As predicted, all conditions revealed FMθ oscillation since 100 ms after trait onset, particularly in a time period at 200–750 ms. According to their topographical distribution (see Supplementary Figure 2 in Supplementary Materials), we selected six frontal–center electrodes (i.e., Fz, F1, F2, FCz, FC1, and FC2) as the ROI of the FMθ for the average power analysis. To compare TF data at each condition and overcome the multiple comparison problem, a random permutation test in ANOVA model was utilized to analyze TF data at a 3- to 30-Hz frequency band within the 200- to 750-ms time windows.

**Figure 5:**
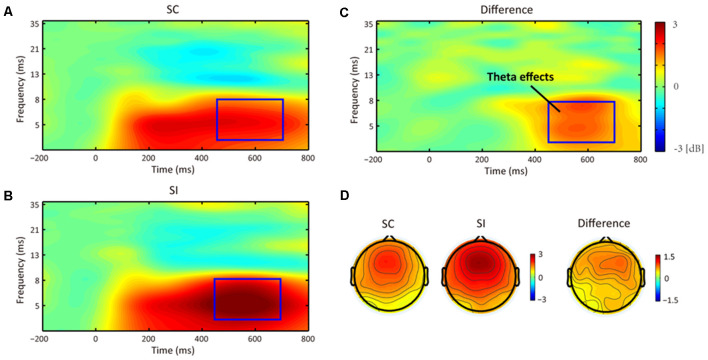
The averaged time-frequency (TF) effects for the stereotype consistent (SC) condition **(A)** and stereotype inconsistent (SI) condition **(B)**, and their difference **(C)** at frontal–center scalp (i.e., the Fz, F1, F2, FCz, FC1, and FC2 electrodes). Compared to the SC condition, the SI condition elicited a significant theta effect (i.e., FMθ) at 450–700 ms. Panel **(D)** shows their topographical distributions.

**Figure 6:**
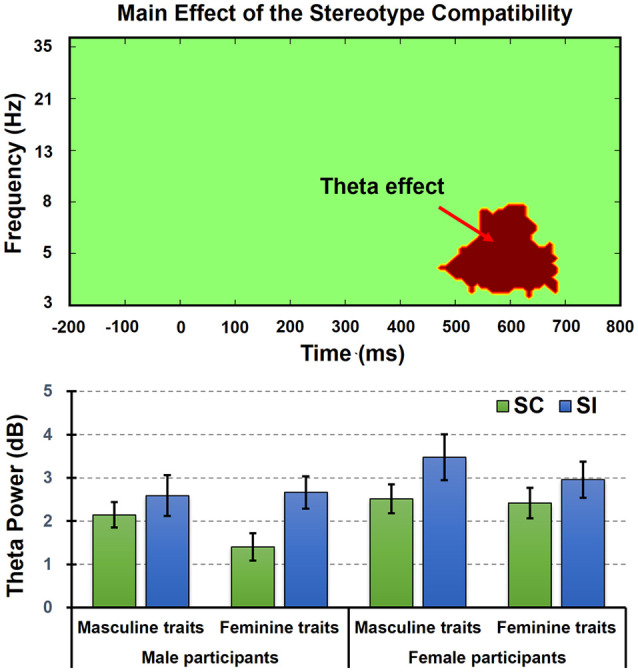
The TF effect revealed by the permutation test in analysis of variance (ANOVA) model. Here we only found a TF effect referring to the main effect of stereotype compatibility at theta band (4–8 Hz) from 450 to 700 ms (upper figure), in which SI conditions elicited stronger frontal–center theta power than SC conditions (lower figure). The column and bar, respectively, denoted the mean and standard error (i.e., mean ± SE).

As results, we only found a TF effect referring to the main effect of stereotype compatibility (see Figures 5, 6). As we predicted, the permutation test indeed revealed a significant cluster at theta band (4–8 Hz) from 450 to 700 ms, in which SI conditions (mean = 2.92 dB, SE = 0.27 dB) elicited stronger frontal–center theta power than SC conditions (mean = 2.12 dB, SE = 0.20 dB). Besides that, no significant main effect or interaction was found.

## Discussion

Using gender names and traits as materials in a modified EAST paradigm, the present study tested the temporal processing of implicit gender stereotyping and further investigated the potential association between the frontal middle theta oscillation and stereotype control.

In line with previous findings (Devine, 1989; Blair and Banaji, 1996; Moskowitz and Li, 2011), our behavioral results showed that SI trials resulted in reduced response accuracies and slower RTs than SC trials, revealing a typical stereotype control effect in EAST. Through ERP measurement, the temporal processing of stereotype control was tested. Here we found conflict-related ERP potentials, such as N400 and LPP, were elicited at a late temporal processing stage and merely showed their susceptibility to conflicts caused by stereotype violation.

The classic N400 is a negative potential usually with a posterior–center scalp distribution. It is sensitive to semantic processes, and its amplitude is usually modulated by violations at the level of semantics or meaning. Previous research has demonstrated that the N400 could reflect the semantic conflicts caused by stereotype violation (e.g., White et al., 2009). In the present study, however, we observed an anterior/frontal N400 (i.e., FN400), which was sensitive to the stereotype compatibility. In fact, the FN400 component is usually seen in memory recognition task (particularly on conceptual priming), but rarely observed in stereotype research (Leynes et al., 2017). Even so, Leynes and Nagovsky (2016) found a FN400 component appeared in a task with contexts of gender stereotype activation. Considering that, the enhanced FN400 amplitude in the SI trials when compared to the SC trials in the present study might reflect a conflict monitoring of conceptual priming on gender stereotype activation.

The LPP usually reflects the cognitive evaluation at the late processing stage. In previous studies, it has been regarded as an index of social expectancy violation. Like N400, it was also sensitive to the stereotype compatibility and commonly showed its enhanced amplitude to SI trials than SC trials (Bartholow and Dickter, 2008; Jia et al., 2012). But in this EAST paradigm, its amplitude was larger at SC condition than at the SI condition. Considering that, it should not index the social expectancy violation in the current study. Instead, because the LPP is also related to the task-irrelevant interference in general conflict control paradigms (Weinberg and Hajcak, 2011), we believed that this stereotype compatibility effect on LPP should reflect a stereotype control process at the late response stage. It was this control process that inhibits the task-irrelevant gender stereotype activation and finally guarantees the color judgment on traits.

Nevertheless, these ERP results (i.e., N400 and LPP effects) also reveal two crucial findings. First, they indeed confirmed our first hypothesis and supported the perspective of the dual-process model, such that the control of implicit gender stereotyping happens at the late temporal processing stage of stereotyping (Devine, 1989; Blair and Banaji, 1996; Moskowitz and Li, 2011). Second, they also indicated that the control of implicit gender stereotyping was not modulated by the experimental manipulations or individual differences in gender factors.

However, early attention-related ERP components such as P2 and N2 were modulated by the gender factors but not stereotype compatibility in the present study. Specifically, the P2 amplitude was modulated by the participant’s gender, in which male participants evoked a larger P2 amplitude, whereas N2 amplitude was modulated by the gender preference of stimuli, in which all participants evoked a larger N2 amplitude to the masculine traits. In previous stereotype studies, P2 and N2 amplitudes were sensitive to racial and gender cues and reflected the social categorization in early attentional processes (Ito and Urland, 2003; Dickter and Bartholow, 2007, 2010). In the present study, although the P2 and N2 effects did not reflect stereotype control processes, they might support a previous attention selection model that P2 and N2 reflect the social categorization (e.g., gender categorization) of gender stereotype activation in early attentional processes (Ito and Urland, 2003; Dickter and Bartholow, 2007). According to this model, the larger P2 amplitude of male participants might indicate that those male participants might input more attentional resource to the gender categorization processes, whereas the larger N2 amplitude to the masculine traits indicated that masculine traits were more attentional focused. This explanation could be supported from the participants’ gender × traits’ gender preference interaction in the behavioral results, in which male participants responded faster to the masculine traits than feminine traits.

Besides that, the most important finding in the current study is the stereotype control process reflected by enhanced frontal theta power. As mentioned, previous research has indicated that the stereotype control shared a similar conflict interference mechanism with the domain-general cognitive control of the prefrontal cortices (Knutson et al., 2007; Cattaneo et al., 2011), whereas the frontal middle theta oscillation could index the domain-general cognitive control mechanism (Nigbur et al., 2011; Cavanagh and Frank, 2014). Based on this knowledge, we predicted that the implicit gender stereotype control processes could be reflected by enhanced frontal middle theta power in the present EAST paradigm. As a result, we observed frontal theta oscillations, respectively, in both SC and SI conditions. They appeared about 100 ms after trait onset and lasted until participant’s responses. Paired comparison on frontal theta power indicated they were indeed sensitive to the stereotype control process. Specifically, the SI conditions when compared to the SC conditions showed reduced response accuracies and slower RTs but elicited enhanced ERSs on frontal theta oscillation at 450–700 ms (i.e., the time window from N400 to LPP). Hence, this result supported our second hypothesis and revealed a novel finding relevant to the frontal middle theta oscillation in stereotype processes.

To the best of our knowledge, our study is the first to demonstrate a robust FMθ oscillation in the processing of implicit gender stereotypes, particularly with the recruitment of stereotype control at a late response stage. In recent studies on general conflict control, the ERS effect on frontal theta power usually shares a time window similar to the N2 or ERN component and reflects the recruitment of executive control at a much early processing stage (Nigbur et al., 2011; Padrão et al., 2015; Brunetti et al., 2019). However, as these studies demonstrated, the FMθ of executive control could vary across different interference situations (e.g., Nigbur et al., 2011). This could explain why the ERS effect on FMθ in our study appears at a much later processing stage. Taken together, this ERS effect on FMθ fits not only with the previous ERP finding on stereotype control (e.g., N400 and LPP effects; Bartholow et al., 2006; White et al., 2009), but is also consistent with the EEG spectral performance of domain-general cognitive control mechanism (e.g., Nigbur et al., 2011; Cavanagh and Frank, 2014). Therefore, our findings provided further evidence that implicit stereotype control relied on domain-general cognitive control regions in prefrontal cortices.

In addition, we also found that the FMθ oscillation was evoked in each condition at the early perception stage (particularly, 200–300 ms after trait onset, which correspond to a time window from the P2 to the N2). Unlike the P2 and N2 amplitude, the power of this frontal middle theta was not sensitive to the implicit stereotype control or gender factors. Compared to the ERP measurements, it should be noted that this theta power was not phase-locked. Thereby, frontal middle theta oscillation at this stage should reflect a different processing mechanism from the gender categorization reflected by the P2 and N2. In fact, previous research has demonstrated that focused attention could also elicit the frontal theta oscillation in DLPFC at an early perceptual stage (Missonnier et al., 2006; Doppelmayr et al., 2008). Considering that, the frontal theta oscillation at the early perception stage might merely reflect initial focused attention to the target traits. Because of its non–phase-locked feature, the frontal theta oscillation might be less sensitive than ERPs (i.e., P2 and N2) to measure the impact factors on early attentional process. However, this explanation is only an interim guess and still needs to be tested in future research.

## Data Availability Statement

The raw data supporting the conclusions of this article will be made available by the authors, without undue reservation.

## Ethics Statement

The studies involving human participants were reviewed and approved by the Ethics Committee of Zhejiang Normal University. The patients/participants provided their written informed consent to participate in this study.

## Author Contributions

LJ: experiment design, data analysis, and draft writing. MC: data collection. BS: draft writing. CW: data analysis. JW: research conception, experiment design, and draft writing. FL: experiment design, and draft writing. All authors contributed to the article and approved the submitted version.

## Conflict of Interest

The authors declare that the research was conducted in the absence of any commercial or financial relationships that could be construed as a potential conflict of interest.

## Supplementary Material

SUPPLEMENTARY TABLE 1 Experimental materials used in the present study.

SUPPLEMENTARY FIGURE 1 The grounded average of time-frequency (TF) decomposition for each condition.

SUPPLEMENTARY FIGURE 2 Topographical distribution of frontal midline theta oscillation for each condition at 450–700 ms.

## References

[B1] Amodio D. M. Devine P. G. Harmon-Jones E. (2008). Individual differences in the regulation of intergroup bias: the role of conflict monitoring and neural signals for control. J. Pers. Soc. Psychol. 94, 60–74. 10.1037/0022-3514.94.1.60 18179318

[B2] Bartholow B. D. Dickter C. L. (2008). A response conflict account of the effects of stereotypes on racial categorization. Soc. Cognit. 26, 314–332. 10.1521/soco.2008.26.3.314

[B3] Bartholow B. D. Dickter C. L. Sestir M. A. (2006). Stereotype activation and control of race bias: cognitive control of inhibition and its impairment by alcohol. J. Pers. Soc. Psychol. 90, 272–287. 10.1037/0022-3514.90.2.272 16536651

[B330] Bartholow B. D. Pearson M. A. Gratton G. Fabiani M. (2003). Effects of alcohol on person perception: A social cognitive neuroscience approach. J. Pers. Soc. Psychol. 85, 627–638. 10.1037/0022-3514.85.4.627 14561117

[B331] Blair I. V. Banaji M. R. (1996). Automatic and controlled processes in stereotype priming. J. Pers. Soc. Psychol. 70, 1142–1163. 10.1037/0022-3514.70.6.114216536651

[B4] Botvinick M. M. Braver T. S. Barch D. M. Carter C. S. Cohen J. D. (2001). Conflict monitoring and cognitive control. Psychol. Rev. 108, 624–652. 10.1037/0033-295x.108.3.624 11488380

[B5] Brunetti M. Zappasodi F. Croce P. Di Matteo R. (2019). Parsing the Flanker task to reveal behavioral and oscillatory correlates of unattended conflict interference. Sci. Rep. 9:13883. 10.1038/s41598-019-50464-x 31554881 PMC6761179

[B6] Cattaneo Z. Mattavelli G. Platania E. Papagno C. (2011). The role of the prefrontal cortex in controlling gender-stereotypical associations: a TMS investigation. NeuroImage 56, 1839–1846. 10.1016/j.neuroimage.2011.02.037 21338690

[B7] Cavanagh J. F. Frank M. J. (2014). Frontal theta as a mechanism for cognitive control. Trends Cogn. Sci. 18, 414–421. 10.1016/j.tics.2014.04.012 24835663 PMC4112145

[B8] Correll J. Urland G. R. Ito T. A. (2006). Event-related potentials and the decision to shoot: the role of threat perception and cognitive control. J. Exp. Soc. Psychol. 42, 120–128. 10.1016/j.jesp.2005.02.006

[B9] De Houwer J. (2006). The extrinsic affective simon task. Exp. Psychol. 50, 77–85. 10.1026//1618-3169.50.2.77 12693192

[B10] Delorme A. Makeig S. (2004). EEGLAB: an open source toolbox for analysis of single-trial EEG dynamics including independent component analysis. J. Neurosci. Methods 134, 9–21. 10.1016/j.jneumeth.2003.10.009 15102499

[B11] Devine P. G. (1989). Stereotypes and prejudice: their automatic and controlled components. J. Pers. Soc. Psychol. 56, 5–18. 10.1037/0022-3514.56.1.5

[B12] Dickter C. L. Bartholow B. D. (2007). Racial ingroup and outgroup attention biases revealed by event-related brain potentials. Soc. Cogn. Affect. Neurosci. 2, 189–198. 10.1093/scan/nsm012 18985140 PMC2569810

[B13] Dickter C. L. Bartholow B. D. (2010). Ingroup categorization and response conflict: interactive effects of target race, flanker compatibility, and infrequency on N2 amplitude. Psychophysiology 47, 596–601. 10.1111/j.1469-8986.2010.00963.x 20136734

[B14] Doppelmayr M. Finkenzeller T. Sauseng P. (2008). Frontal midline theta in the pre-shot phase of rifle shooting: differences between experts and novices. Neuropsychologia 46, 1463–1467. 10.1016/j.neuropsychologia.2007.12.026 18280523

[B15] Hamm J. P. Ethridge L. E. Shapiro J. R. Stevens M. C. Boutros N. N. Summerfelt A. T.. (2012). Spatiotemporal and frequency domain analysis of auditory paired stimuli processing in schizophrenia and bipolar disorder with psychosis. Psychophysiology 49, 522–530. 10.1111/j.1469-8986.2011.01327.x 22176721 PMC3309114

[B16] Hamm J. P. Gilmore C. S. Picchetti N. A. M. Sponheim S. R. Clementz B. A. (2011). Abnormalities of neuronal oscillations and temporal integration to low- and high-frequency auditory stimulation in schizophrenia. Biol. Psychiatry 69, 989–996. 10.1016/j.biopsych.2010.11.021 21216392 PMC3174270

[B17] Ito T. A. Urland G. R. (2003). Race and gender on the brain: electrocortical measures of attention to the race and gender of multiply categorizable individuals. J. Pers. Soc. Psychol. 85, 616–626. 10.1037/0022-3514.85.4.616 14561116

[B18] Jia L. Dickter C. L. Luo J. Xiao X. Yang Q. Lei M.. (2012). Different brain mechanisms between stereotype activation and application: evidence from an ERP study. Int. J. Psychol. 47, 58–66. 10.1080/00207594.2011.580348 22047000

[B19] Knutson K. M. Mah L. Manly C. F. Grafman J. (2007). Neural correlates of automatic beliefs about gender and race. Hum. Brain Mapp. 28, 915–930. 10.1002/hbm.20320 17133388 PMC6871386

[B21] Leynes P. A. Bruett H. Krizan J. Veloso A. (2017). What psychological process is reflected in the FN400 event-related potential component? Brain Cogn. 113, 142–154. 10.1016/j.bandc.2017.02.004 28235696

[B20] Leynes P. A. Nagovsky I. (2016). Influence of encoding focus and stereotypes on source monitoring event-related-potentials. Brain Res. 1630, 171–182. 10.1016/j.brainres.2015.11.017 26604033

[B22] Liu Z.-X. Woltering S. Lewis M. D. (2014). Developmental change in EEG theta activity in the medial prefrontal cortex during response control. NeuroImage 85, 873–887. 10.1016/j.neuroimage.2013.08.054 24007804

[B23] Ma Q. Shu L. Wang X. Dai S. Che H. (2008). Error-related negativity varies with the activation of gender stereotypes. Neurosci. Lett. 442, 186–189. 10.1016/j.neulet.2008.06.080 18619519

[B24] MacDonald A. W. III. Cohen J. D. Stenger V. A. Carter C. S. (2000). Dissociating the role of the dorsolateral prefrontal and anterior cingulate cortex in cognitive control. Science 288, 1835–1838. 10.1126/science.288.5472.1835 10846167

[B25] Macrae C. N. Bodenhausen G. V. (2000). Social cognition: thinking categorically about others. Annu. Rev. Psychol. 51, 93–120. 10.1146/annurev.psych.51.1.93 10751966

[B26] Missonnier P. Deiber M.-P. Gold G. Millet P. Gex-Fabry Pun M. Fazio-Costa L.. (2006). Frontal theta event-related synchronization: comparison of directed attention and working memory load effects. J. Neural Transm. 113, 1477–1486. 10.1007/s00702-005-0443-9 16604309

[B27] Moskowitz G. B. Li P. (2011). Egalitarian goals trigger stereotype inhibition: a proactive form of stereotype control. J. Exp. Soc. Psychol. 47, 103–116. 10.1016/j.jesp.2010.08.014

[B28] Nigbur R. Ivanova G. Stürmer B. (2011). Theta power as a marker for cognitive interference. Clin. Neurophysiol. 122, 2185–2194. 10.1016/j.clinph.2011.03.030 21550845

[B29] Padrão G. Rodriguez-Herreros B. Pérez Zapata L. Rodriguez-Fornells A. (2015). Exogenous capture of medial-frontal oscillatory mechanisms by unattended conflicting information. Neuropsychologia 75, 458–468. 10.1016/j.neuropsychologia.2015.07.004 26151855

[B30] van Veen V. Carter C. S. (2005). Separating semantic conflict and response conflict in the Stroop task: a functional MRI study. NeuroImage 27, 497–504. 10.1016/j.neuroimage.2005.04.042 15964208

[B31] Weinberg A. Hajcak G. (2011). The late positive potential predicts subsequent interference with target processing. J. Cogn. Neurosci. 23, 2994–3007. 10.1162/jocn.2011.21630 21268668

[B32] Wentura D. (2010). The extrinsic affective Simon task as an instrument for indirect assessment of prejudice. Eur. J. Soc. Psychol. 38, 1033–1043. 10.1002/ejsp.536

[B33] White K. R. Crites S. L. Jr. Taylor J. H. Corral G. (2009). Wait, what? Assessing stereotype incongruities using the N400 ERP component. Soc. Cogn. Affect. Neurosci. 4, 191–198. 10.1093/scan/nsp004 19270040 PMC2686231

