# Supplementary material for: Frontal Theta Oscillation as a Mechanism for Implicit Gender Stereotype Control: Electrophysiological Evidence From an Extrinsic Affective Simon Task
Source: Front Hum Neurosci. 2020 Dec 17;14:573187. doi: 10.3389/fnhum.2020.573187 (PMC7773647; doi:10.3389/fnhum.2020.573187)
Supplement: SUPPLEMENTARY TABLE 1 — Experimental materials used in the present study. [file Table_1.DOCX]

Supplementary Materials

# Supplementary Figures and Tables

**1.1 Experimental materials**

**Table S1: Experimental materials used in the present study**

| Male names | 李飞 赵虎 刘伟 郑斌 王峰 雷励 高军 孙祥 邹彪 薛磊 韩鹏 林涛 |
| --- | --- |
| Female names | 夏雪 柳芸 马芳 陈丽 朱萍 何雯 叶颖 贺玲 杨慧 杜妍 曾倩 徐莉 |
| Masculine traits | 彪悍 阳刚 骁勇 英俊 粗犷 强壮 魁梧 刚毅 挺拔 健硕 帅气 俊朗 |
| Feminine traits | 柔弱 文静 娟秀 娇俏 娴雅 温婉 乖巧 贤惠 温柔 窈窕 婀娜 端庄 |

**^a^** All gender traits are chosen from a 9-point (1=very masculine, 5= gender neutral, 9=very feminine) gender role scale normed on an independent sample of 37 Chinese volunteers in the Southwest University.

**^b^** Twelve masculine traits in Chinese language are positive adjectives mainly describing masculine features such as heroic, intrepid, strong, aggressive, and rugged. In contrast, twelve feminine traits n Chinese language are positive adjectives mainly describing feminine features such as attractive, lovely, elegant, and graceful.

**^c^** All names are chosen from internet. All of them passed a quick gender categorization by each participant before the former experiment.

**1.2 Time-frequency (TF) results**


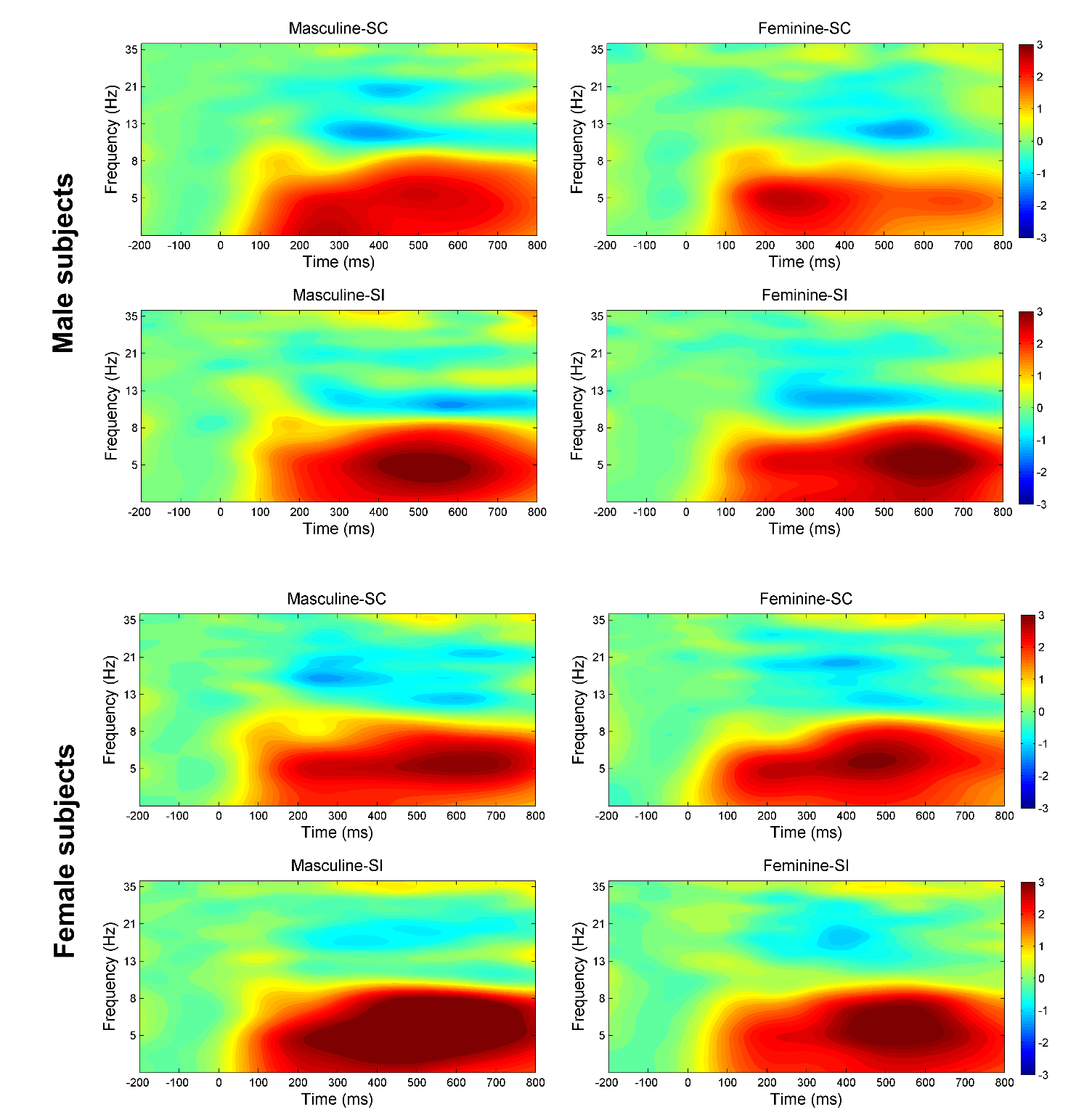


**Figure S1: The grounded average of TF decomposition for each condition**


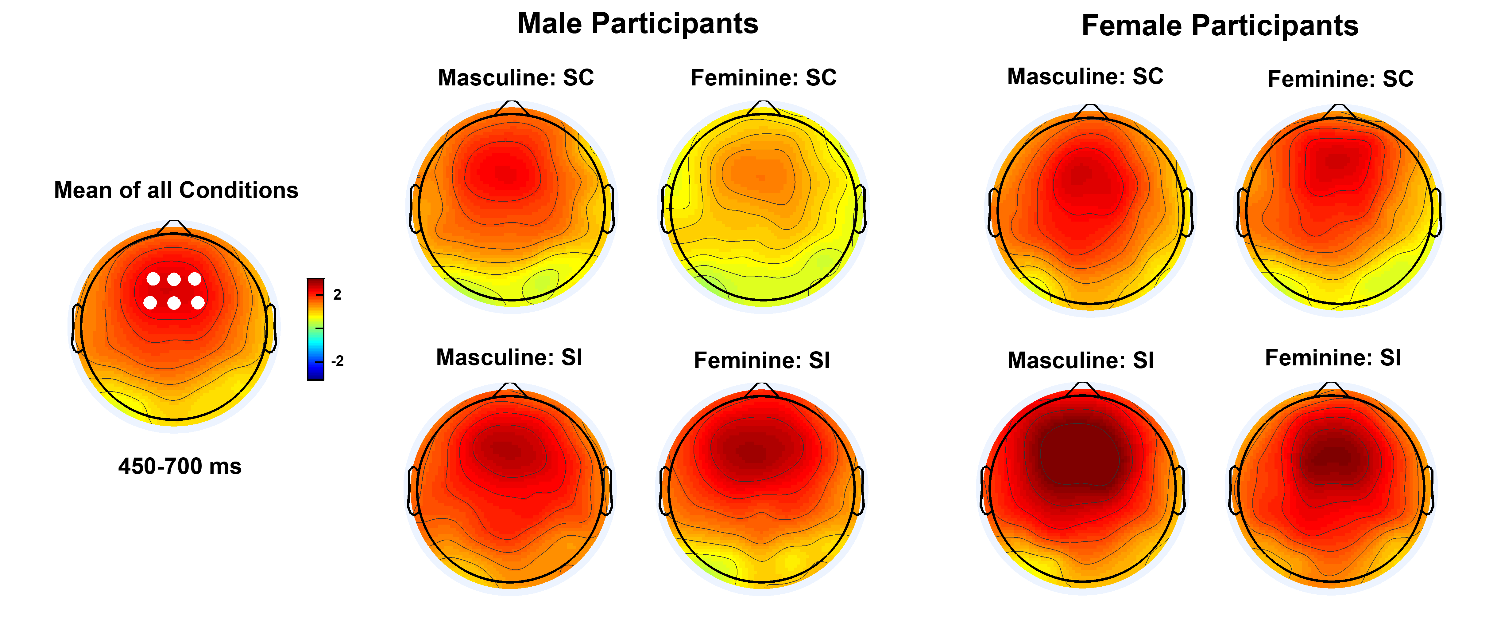


**Figure S2: Topographical distribution of frontal midline theta oscillation for each condition at 450-700 ms**
